# *N*-[2-(6-Methyl-4-oxo-4*H*-chromen-3-yl)-4-oxothia­zolidin-3-yl]furan-2-carbox­amide *N*,*N*-dimethyl­formamide solvate

**DOI:** 10.1107/S1600536809029572

**Published:** 2009-07-29

**Authors:** Pei-Liang Zhao, Zhong-Zhen Zhou

**Affiliations:** a Department of Chemistry, Pharmaceutical Sciences, Southern Medical Universtiy, Guangzhou 510515, People’s Republic of China

## Abstract

The title mol­ecule, C_18_H_14_N_2_O_5_S·C_3_H_7_NO, comprises of a carboxamide group bonded to a furan ring and a distorted envelope-shaped 4-oxothia­zolidin-3-yl group which is connected to a substituted 6-methyl-4-oxo-4*H*-chromen-3-yl group. Extensive strong N—H⋯O and weak C—H⋯O inter­molecular hydrogen-bonding inter­actions occur between dimethyl­formamide (DMF), the crystallizing solvent, and the various heterocyclic groups within the compound, as well as additional weak C—H⋯O inter­actions between the heterocyclic groups themselves. The carboxyl group of the DMF solvent mol­ecule forms a trifurcated (four-center) acceptor hydrogen-bond inter­action with the carboxamide, furan and 6-methyl-4-oxo-4*H*-chromen-3-yl groups. The dihedral angles between the planar chromone group [maximum deviation = 0.0377 (18)°] and those of the furan and 4-oxothia­zolidin-3-yl groups are 89.4 (6) and 78.5 (1)°, respectively.

## Related literature

For related structures, see: Zhou *et al.* (2005 ▶). For the preparation of the title compound, see: Zhou *et al.* (2008 ▶). For general background to glycoluril and its derivatives, see: Maliar *et al.* (2004 ▶), Zhou *et al.* (2007 ▶). For puckering parameters, see: Cremer & Pople (1975 ▶). 
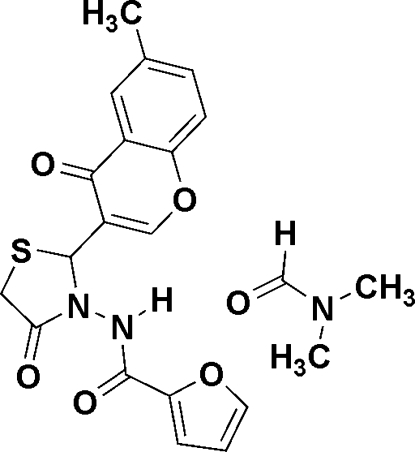


## Experimental

### 

#### Crystal data

C_18_H_14_N_2_O_5_S·C_3_H_7_NO
*M* *_r_* = 443.48
Triclinic, 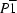

*a* = 8.4141 (1) Å
*b* = 11.5676 (14) Å
*c* = 11.8382 (14) Å
α = 87.138 (1)°
β = 70.503 (2)°
γ = 78.419 (2)°
*V* = 1063.86 (18) Å^3^
*Z* = 2
Mo *K*α radiation
μ = 0.20 mm^−1^
*T* = 292 K
0.30 × 0.20 × 0.20 mm

#### Data collection

Bruker SMART APEX CCD area-detector diffractometer
Absorption correction: multi-scan (*SADABS*; Sheldrick, 2008 ▶) *T*_min_ = 0.934, *T*_max_ = 0.962
7900 measured reflections
4568 independent reflections
3163 reflections with *I* > 2σ(*I*)
*R*_int_ = 0.043

#### Refinement

*R*[*F* ^2^ > 2σ(*F* ^2^)] = 0.049
*wR*(*F* ^2^) = 0.130
*S* = 0.96
4568 reflections
289 parameters
H atoms treated by a mixture of independent and constrained refinement
Δρ_max_ = 0.25 e Å^−3^
Δρ_min_ = −0.17 e Å^−3^

### 

Data collection: *SMART* (Bruker, 2001 ▶); cell refinement: *SAINT* (Bruker, 2001 ▶); data reduction: *SAINT*; program(s) used to solve structure: *SHELXS97* (Sheldrick, 2008 ▶); program(s) used to refine structure: *SHELXL97* (Sheldrick, 2008 ▶); molecular graphics: *PLATON* (Spek, 2009 ▶); software used to prepare material for publication: *SHELXL97*.

## Supplementary Material

Crystal structure: contains datablocks global, I. DOI: 10.1107/S1600536809029572/jj2002sup1.cif

Structure factors: contains datablocks I. DOI: 10.1107/S1600536809029572/jj2002Isup2.hkl

Additional supplementary materials:  crystallographic information; 3D view; checkCIF report

## Figures and Tables

**Table 1:** Hydrogen-bond geometry (Å, °)

*D*—H⋯*A*	*D*—H	H⋯*A*	*D*⋯*A*	*D*—H⋯*A*
N2—H2⋯O6	0.77 (2)	2.10 (2)	2.856 (2)	169 (2)
C4—H4⋯O6^i^	0.93	2.50	3.421 (3)	172
C10—H10⋯O4^ii^	0.93	2.49	3.332 (2)	151
C11—H11⋯O5^ii^	0.98	2.50	3.267 (2)	135 (1)
C13—H13*B*⋯O3^iii^	0.97	2.55	3.441 (2)	153
C16—H16⋯O6	0.93	2.36	3.193 (3)	150
C18—H18⋯O3^iv^	0.93	2.47	3.342 (3)	157
C20—H20*C*⋯O1^v^	0.96	2.46	3.361 (3)	157

Symmetry codes: (i) 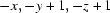; (ii) 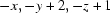; (iii) 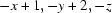; (iv) 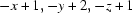; (v) 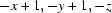.

**Fig. 1.**
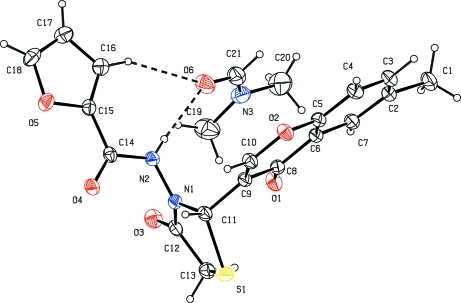
The asymmetric unit of the title molecule with displacement ellipsoids for the non-hydrogen atoms drawn at the 30% probability level. Hydrogen bonds are drawn as dashed lines.

**Fig. 2.**
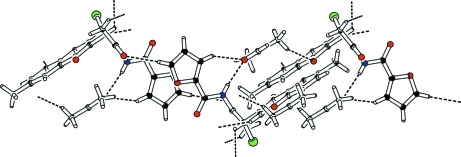
The packing of the title molecule, showing one layer of molecules connected by strong N—H···O, and weak C—H···O intermolecular hydrogen bonds, and weak π-π stacking interactions.

## supplementary crystallographic information

### Comment

The tri-substituted chromone (1,4-benzopyrone) pharmacophore is an important structural element in medicinal chemistry, and shows a broad spectrum of pharmacological activities (Maliar *et al.* (2004), Zhou *et al.* (2007)). Hence, we were curious to explore the family of biheterocyclic compounds that contain both the thiazolidinone and chromone pharmacophores, with a view to discovering novel lead structures for the development of antifungal agents.

The title molecule is comprised of a carboxamide group bonded to a furan ring and a distorted envelope shaped (Cremer & Pople, 1975) 4-oxothiazolidin-3-yl group (Q(2) = 0.1878 (18) Å, Phi(2) = 188.3 (6)°; for an ideal envelope Phi(2) = k *x* 36) which is connected to a substituted 6-methyl-4-oxo-4*H*-chromen-3-yl group (Fig. 1).  Extensive strong N2—H2···O6 and weak C16—H16···O6, C20—H20···O6, hydrogen bonding intermolecular interactions occur between dimethylformamide (DMF), the crystallizing solvent, and the various heterocyclic groups within the compound as well as additional weak C—H···O interactions between the heterocyclic groups themselves (Table 1, Fig. 2).  The carboxyl group of the DMF solvent forms a trifurcated (4-center) acceptor hydrogen bond interaction with the carboxamide, furan and 6-methyl-4-oxo-4*H*-chromen-3-yl groups.  The dihedral angle between the planar chromone group (max deviation = 0.0377 (18)°) and that of the furan and 4-oxothiazolidin-3-yl groups is 89.4 (6)° and 78.5 (1)°, respectively.  Crystal packing is also stabilized by π-π stacking interactions (*Cg*2—*Cg*2 = 3.8378 (14) Å; 1 - *x*, 2 - *y*, 1 - *z*; *Cg*2 is the centroid of the O5/C15—C18 ring).

### Experimental

The title compound was synthesized according to the procedure reported (Zhou *et al.*, 2008).  Crystals appropriate for X-ray data collection were obtained by slow evaporation of the DMF solution at 293 K.

### Refinement

All H atoms were initially located in a difference Fourier map.  The methyl H atoms were constrained to an ideal geometry, with C—H distances of 0.96 Å, and *U*_iso_(H) = 1.49–1.50 *U*_eq_C.  Each group was allowed to rotate freely about its C–C bond. All other H atoms were placed in geometrically idealized positions and constrained to ride on their parent atoms, with C–H = 0.93–0.98 Å, N–H = 0.77Å and *U*_iso_(H) = 1.19–1.20*U*_eq_(C,*N*).)

### Crystal data

**Table:**

C_18_H_14_N_2_O_5_S·C_3_H_7_NO	*Z* = 2
*M**_r_* = 443.48	*F*(000) = 464
Triclinic, *P*1	*D*_x_ = 1.384 Mg m^−^^3^
Hall symbol: -P 1	Mo *K*α radiation, λ = 0.71073 Å
*a* = 8.4141 (1) Å	Cell parameters from 2421 reflections
*b* = 11.5676 (14) Å	θ = 2.5–26.7°
*c* = 11.8382 (14) Å	µ = 0.20 mm^−^^1^
α = 87.138 (1)°	*T* = 292 K
β = 70.503 (2)°	Block, colorless
γ = 78.419 (2)°	0.30 × 0.20 × 0.20 mm
*V* = 1063.86 (18) Å^3^	

### Data collection

**Table:**

Bruker SMART APEX CCD area-detector diffractometer	4568 independent reflections
Radiation source: fine-focus sealed tube	3163 reflections with *I* > 2σ(*I*)
graphite	*R*_int_ = 0.043
φ and ω scans	θ_max_ = 27.0°, θ_min_ = 1.8°
Absorption correction: multi-scan (*SADABS*; Sheldrick, 2008)	*h* = −10→10
*T*_min_ = 0.934, *T*_max_ = 0.962	*k* = −13→14
7900 measured reflections	*l* = −15→15

### Refinement

**Table:**

Refinement on *F*^2^	Primary atom site location: structure-invariant direct methods
Least-squares matrix: full	Secondary atom site location: difference Fourier map
*R*[*F*^2^ > 2σ(*F*^2^)] = 0.049	Hydrogen site location: inferred from neighbouring sites
*wR*(*F*^2^) = 0.130	H atoms treated by a mixture of independent and constrained refinement
*S* = 0.96	*w* = 1/[σ^2^(*F*_o_^2^) + (0.0676*P*)^2^] where *P* = (*F*_o_^2^ + 2*F*_c_^2^)/3
4568 reflections	(Δ/σ)_max_ < 0.001
289 parameters	Δρ_max_ = 0.25 e Å^−^^3^
0 restraints	Δρ_min_ = −0.16 e Å^−^^3^

### Special details

**Table:**

Geometry. All e.s.d.'s (except the e.s.d. in the dihedral angle between two l.s. planes) are estimated using the full covariance matrix. The cell e.s.d.'s are taken into account individually in the estimation of e.s.d.'s in distances, angles and torsion angles; correlations between e.s.d.'s in cell parameters are only used when they are defined by crystal symmetry. An approximate (isotropic) treatment of cell e.s.d.'s is used for estimating e.s.d.'s involving l.s. planes.
Refinement. Refinement of *F*^2^ against ALL reflections. The weighted *R*-factor *wR* and goodness of fit *S* are based on *F*^2^, conventional *R*-factors *R* are based on *F*, with *F* set to zero for negative *F*^2^. The threshold expression of *F*^2^ > σ(*F*^2^) is used only for calculating *R*-factors(gt) *etc*. and is not relevant to the choice of reflections for refinement. *R*-factors based on *F*^2^ are statistically about twice as large as those based on *F*, and *R*- factors based on ALL data will be even larger.

### Fractional atomic coordinates and isotropic or equivalent isotropic displacement parameters (Å^2^)

**Table:**

	*x*	*y*	*z*	*U*_iso_*/*U*_eq_	
C1	0.2135 (3)	0.19985 (18)	0.1714 (2)	0.0722 (7)	
H1A	0.1411	0.1794	0.1309	0.108*	
H1B	0.2338	0.1381	0.2251	0.108*	
H1C	0.3210	0.2097	0.1135	0.108*	
C2	0.1263 (3)	0.31350 (16)	0.24164 (17)	0.0519 (5)	
C3	−0.0211 (3)	0.31675 (18)	0.34263 (19)	0.0580 (6)	
H3	−0.0598	0.2469	0.3680	0.070*	
C4	−0.1099 (3)	0.41904 (17)	0.40512 (18)	0.0542 (5)	
H4	−0.2082	0.4192	0.4713	0.065*	
C5	−0.0502 (2)	0.52272 (16)	0.36769 (16)	0.0445 (4)	
C6	0.0963 (2)	0.52416 (15)	0.26977 (15)	0.0420 (4)	
C7	0.1832 (3)	0.41704 (16)	0.20822 (16)	0.0484 (5)	
H7	0.2825	0.4164	0.1427	0.058*	
C8	0.1533 (2)	0.63617 (16)	0.22977 (15)	0.0427 (4)	
C9	0.0433 (2)	0.73858 (15)	0.30060 (15)	0.0414 (4)	
C10	−0.0936 (2)	0.72643 (16)	0.39626 (16)	0.0467 (5)	
H10	−0.1573	0.7941	0.4410	0.056*	
C11	0.0758 (2)	0.86128 (16)	0.26908 (16)	0.0443 (4)	
H11	−0.005	0.9161	0.3323	0.053*	
C12	0.3611 (3)	0.89041 (15)	0.14565 (16)	0.0469 (5)	
C13	0.2766 (3)	0.90147 (18)	0.05140 (17)	0.0573 (5)	
H13A	0.3236	0.8335	−0.0027	0.069*	
H13B	0.2968	0.9719	0.0052	0.069*	
C14	0.2690 (2)	0.93551 (16)	0.43651 (15)	0.0415 (4)	
C15	0.3436 (2)	0.89915 (16)	0.53174 (15)	0.0430 (4)	
C16	0.4445 (3)	0.80082 (19)	0.55371 (19)	0.0593 (5)	
H16	0.4843	0.7310	0.5086	0.071*	
C17	0.4781 (3)	0.8243 (2)	0.6579 (2)	0.0678 (6)	
H17	0.5443	0.7728	0.6952	0.081*	
C18	0.3975 (3)	0.9337 (2)	0.69290 (19)	0.0650 (6)	
H18	0.3986	0.9716	0.7600	0.078*	
C19	0.7238 (4)	0.6242 (3)	0.1424 (3)	0.1048 (10)	
H19A	0.6655	0.6544	0.0867	0.157*	
H19B	0.8457	0.6078	0.1013	0.157*	
H19C	0.6968	0.6818	0.2050	0.157*	
C20	0.7468 (4)	0.4095 (3)	0.1255 (3)	0.1092 (11)	
H20A	0.7150	0.3444	0.1756	0.164*	
H20B	0.8696	0.4012	0.0976	0.164*	
H20C	0.7074	0.4105	0.0581	0.164*	
C21	0.5459 (3)	0.5218 (2)	0.2970 (2)	0.0710 (6)	
H21	0.5136	0.4507	0.3256	0.085*	
N1	0.2489 (2)	0.87432 (13)	0.25530 (13)	0.0442 (4)	
N2	0.3049 (2)	0.85021 (14)	0.35237 (14)	0.0476 (4)	
H2	0.361 (3)	0.7885 (19)	0.351 (2)	0.057*	
N3	0.6696 (3)	0.51810 (17)	0.19314 (17)	0.0676 (5)	
O1	0.28274 (17)	0.64100 (11)	0.14324 (11)	0.0563 (4)	
O2	−0.14547 (16)	0.62410 (11)	0.43227 (11)	0.0507 (3)	
O3	0.51047 (19)	0.89574 (13)	0.12709 (12)	0.0630 (4)	
O4	0.18311 (18)	1.03252 (11)	0.43361 (11)	0.0534 (4)	
O5	0.31260 (18)	0.98314 (12)	0.61700 (12)	0.0576 (4)	
O6	0.4692 (2)	0.60997 (13)	0.35973 (14)	0.0764 (5)	
S1	0.04994 (8)	0.91011 (5)	0.12526 (5)	0.0628 (2)	

### Atomic displacement parameters (Å^2^)

**Table:**

	*U*^11^	*U*^22^	*U*^33^	*U*^12^	*U*^13^	*U*^23^
C1	0.1061 (19)	0.0405 (12)	0.0708 (15)	−0.0139 (12)	−0.0301 (14)	−0.0033 (11)
C2	0.0703 (14)	0.0380 (11)	0.0530 (11)	−0.0120 (10)	−0.0274 (10)	0.0036 (9)
C3	0.0755 (15)	0.0429 (12)	0.0618 (13)	−0.0232 (11)	−0.0255 (11)	0.0115 (10)
C4	0.0597 (13)	0.0518 (12)	0.0518 (11)	−0.0213 (10)	−0.0150 (10)	0.0116 (10)
C5	0.0506 (11)	0.0418 (10)	0.0418 (10)	−0.0112 (9)	−0.0154 (8)	0.0037 (8)
C6	0.0508 (11)	0.0376 (10)	0.0395 (9)	−0.0110 (8)	−0.0163 (8)	0.0027 (8)
C7	0.0585 (12)	0.0419 (10)	0.0436 (10)	−0.0105 (9)	−0.0152 (9)	0.0012 (8)
C8	0.0508 (11)	0.0412 (10)	0.0370 (9)	−0.0129 (9)	−0.0136 (8)	0.0021 (8)
C9	0.0510 (11)	0.0369 (10)	0.0365 (9)	−0.0107 (8)	−0.0134 (8)	0.0014 (8)
C10	0.0516 (11)	0.0379 (10)	0.0460 (10)	−0.0071 (9)	−0.0109 (9)	−0.0015 (8)
C11	0.0529 (11)	0.0358 (10)	0.0403 (10)	−0.0075 (9)	−0.0110 (9)	−0.0008 (8)
C12	0.0619 (13)	0.0316 (10)	0.0436 (10)	−0.0142 (9)	−0.0098 (9)	0.0003 (8)
C13	0.0808 (15)	0.0505 (12)	0.0415 (10)	−0.0224 (11)	−0.0167 (10)	0.0060 (9)
C14	0.0460 (10)	0.0384 (10)	0.0368 (9)	−0.0152 (8)	−0.0053 (8)	−0.0005 (8)
C15	0.0456 (10)	0.0449 (11)	0.0371 (9)	−0.0163 (9)	−0.0071 (8)	−0.0004 (8)
C16	0.0659 (14)	0.0550 (13)	0.0594 (13)	−0.0103 (11)	−0.0244 (11)	−0.0015 (10)
C17	0.0738 (16)	0.0729 (16)	0.0672 (14)	−0.0154 (13)	−0.0374 (12)	0.0094 (12)
C18	0.0711 (15)	0.0889 (18)	0.0464 (12)	−0.0280 (14)	−0.0270 (11)	0.0023 (12)
C19	0.118 (3)	0.101 (2)	0.0788 (19)	−0.0156 (19)	−0.0172 (18)	0.0177 (17)
C20	0.137 (3)	0.094 (2)	0.0798 (19)	0.016 (2)	−0.0315 (19)	−0.0293 (17)
C21	0.0858 (18)	0.0557 (15)	0.0702 (16)	−0.0094 (13)	−0.0262 (14)	−0.0010 (12)
N1	0.0563 (10)	0.0408 (9)	0.0377 (8)	−0.0143 (7)	−0.0156 (7)	0.0006 (7)
N2	0.0655 (11)	0.0351 (9)	0.0426 (8)	−0.0052 (8)	−0.0206 (8)	−0.0017 (7)
N3	0.0767 (13)	0.0657 (12)	0.0566 (11)	0.0029 (10)	−0.0263 (10)	−0.0027 (10)
O1	0.0622 (9)	0.0481 (8)	0.0469 (7)	−0.0175 (7)	0.0017 (7)	−0.0035 (6)
O2	0.0532 (8)	0.0426 (8)	0.0469 (7)	−0.0117 (6)	−0.0030 (6)	0.0020 (6)
O3	0.0613 (10)	0.0649 (10)	0.0587 (9)	−0.0208 (8)	−0.0099 (7)	0.0031 (7)
O4	0.0688 (9)	0.0368 (7)	0.0531 (8)	−0.0047 (7)	−0.0206 (7)	−0.0056 (6)
O5	0.0661 (9)	0.0616 (9)	0.0465 (7)	−0.0110 (7)	−0.0197 (7)	−0.0101 (7)
O6	0.0965 (13)	0.0516 (9)	0.0663 (10)	0.0032 (9)	−0.0167 (9)	−0.0069 (8)
S1	0.0733 (4)	0.0633 (4)	0.0590 (3)	−0.0192 (3)	−0.0307 (3)	0.0198 (3)

### Geometric parameters (Å, °)

**Table:**

C1—C2	1.508 (3)	C13—S1	1.797 (2)
C1—H1A	0.9600	C13—H13A	0.9700
C1—H1B	0.9600	C13—H13B	0.9700
C1—H1C	0.9600	C14—O4	1.212 (2)
C2—C7	1.370 (2)	C14—N2	1.354 (2)
C2—C3	1.401 (3)	C14—C15	1.470 (3)
C3—C4	1.367 (3)	C15—C16	1.347 (3)
C3—H3	0.9300	C15—O5	1.361 (2)
C4—C5	1.388 (2)	C16—C17	1.404 (3)
C4—H4	0.9300	C16—H16	0.9300
C5—O2	1.380 (2)	C17—C18	1.325 (3)
C5—C6	1.384 (2)	C17—H17	0.9300
C6—C7	1.404 (3)	C18—O5	1.363 (2)
C6—C8	1.473 (2)	C18—H18	0.9300
C7—H7	0.9300	C19—N3	1.430 (3)
C8—O1	1.230 (2)	C19—H19A	0.9600
C8—C9	1.450 (2)	C19—H19B	0.9600
C9—C10	1.344 (2)	C19—H19C	0.9600
C9—C11	1.504 (2)	C20—N3	1.438 (3)
C10—O2	1.344 (2)	C20—H20A	0.9600
C10—H10	0.9300	C20—H20B	0.9600
C11—N1	1.448 (2)	C20—H20C	0.9600
C11—S1	1.8349 (19)	C21—O6	1.224 (3)
C11—H11	0.9800	C21—N3	1.315 (3)
C12—O3	1.216 (2)	C21—H21	0.9300
C12—N1	1.356 (2)	N1—N2	1.378 (2)
C12—C13	1.498 (3)	N2—H2	0.77 (2)
			
C2—C1—H1A	109.5	C12—C13—H13B	110.1
C2—C1—H1B	109.5	S1—C13—H13B	110.1
H1A—C1—H1B	109.5	H13A—C13—H13B	108.4
C2—C1—H1C	109.5	O4—C14—N2	123.15 (17)
H1A—C1—H1C	109.5	O4—C14—C15	123.24 (16)
H1B—C1—H1C	109.5	N2—C14—C15	113.61 (17)
C7—C2—C3	117.65 (18)	C16—C15—O5	109.77 (17)
C7—C2—C1	122.10 (19)	C16—C15—C14	134.79 (17)
C3—C2—C1	120.22 (18)	O5—C15—C14	115.37 (16)
C4—C3—C2	122.26 (18)	C15—C16—C17	106.6 (2)
C4—C3—H3	118.9	C15—C16—H16	126.7
C2—C3—H3	118.9	C17—C16—H16	126.7
C3—C4—C5	118.60 (18)	C18—C17—C16	106.9 (2)
C3—C4—H4	120.7	C18—C17—H17	126.5
C5—C4—H4	120.7	C16—C17—H17	126.5
O2—C5—C6	121.90 (15)	C17—C18—O5	110.6 (2)
O2—C5—C4	116.58 (16)	C17—C18—H18	124.7
C6—C5—C4	121.52 (18)	O5—C18—H18	124.7
C5—C6—C7	117.91 (16)	N3—C19—H19A	109.5
C5—C6—C8	120.29 (16)	N3—C19—H19B	109.5
C7—C6—C8	121.76 (16)	H19A—C19—H19B	109.5
C2—C7—C6	122.04 (18)	N3—C19—H19C	109.5
C2—C7—H7	119.0	H19A—C19—H19C	109.5
C6—C7—H7	119.0	H19B—C19—H19C	109.5
O1—C8—C9	123.67 (16)	N3—C20—H20A	109.5
O1—C8—C6	122.18 (17)	N3—C20—H20B	109.5
C9—C8—C6	114.14 (15)	H20A—C20—H20B	109.5
C10—C9—C8	120.52 (16)	N3—C20—H20C	109.5
C10—C9—C11	117.78 (16)	H20A—C20—H20C	109.5
C8—C9—C11	121.69 (15)	H20B—C20—H20C	109.5
O2—C10—C9	125.11 (17)	O6—C21—N3	126.2 (2)
O2—C10—H10	117.4	O6—C21—H21	116.9
C9—C10—H10	117.4	N3—C21—H21	116.9
N1—C11—C9	113.96 (16)	C12—N1—N2	120.45 (16)
N1—C11—S1	103.67 (11)	C12—N1—C11	120.75 (16)
C9—C11—S1	113.20 (12)	N2—N1—C11	117.91 (14)
N1—C11—H11	109.0	C14—N2—N1	119.57 (15)
C9—C11—H11	109.0	C14—N2—H2	125.4 (17)
S1—C11—H11	109.0	N1—N2—H2	114.9 (17)
O3—C12—N1	124.01 (19)	C21—N3—C19	120.1 (2)
O3—C12—C13	124.74 (17)	C21—N3—C20	121.3 (2)
N1—C12—C13	111.25 (17)	C19—N3—C20	118.6 (2)
C12—C13—S1	108.00 (13)	C10—O2—C5	117.98 (14)
C12—C13—H13A	110.1	C15—O5—C18	106.03 (16)
S1—C13—H13A	110.1	C13—S1—C11	93.51 (9)
			
C7—C2—C3—C4	1.8 (3)	N2—C14—C15—C16	2.3 (3)
C1—C2—C3—C4	−176.30 (19)	O4—C14—C15—O5	−1.2 (2)
C2—C3—C4—C5	−0.8 (3)	N2—C14—C15—O5	178.98 (14)
C3—C4—C5—O2	178.99 (17)	O5—C15—C16—C17	0.1 (2)
C3—C4—C5—C6	−0.3 (3)	C14—C15—C16—C17	177.0 (2)
O2—C5—C6—C7	−178.88 (16)	C15—C16—C17—C18	−0.1 (2)
C4—C5—C6—C7	0.4 (3)	C16—C17—C18—O5	0.1 (3)
O2—C5—C6—C8	−1.3 (3)	O3—C12—N1—N2	−7.0 (3)
C4—C5—C6—C8	178.01 (17)	C13—C12—N1—N2	173.40 (15)
C3—C2—C7—C6	−1.7 (3)	O3—C12—N1—C11	−175.98 (17)
C1—C2—C7—C6	176.35 (18)	C13—C12—N1—C11	4.4 (2)
C5—C6—C7—C2	0.6 (3)	C9—C11—N1—C12	109.33 (18)
C8—C6—C7—C2	−176.91 (17)	S1—C11—N1—C12	−14.16 (19)
C5—C6—C8—O1	180.00 (18)	C9—C11—N1—N2	−59.9 (2)
C7—C6—C8—O1	−2.5 (3)	S1—C11—N1—N2	176.57 (11)
C5—C6—C8—C9	−0.4 (2)	O4—C14—N2—N1	2.9 (3)
C7—C6—C8—C9	177.10 (16)	C15—C14—N2—N1	−177.28 (14)
O1—C8—C9—C10	−178.07 (19)	C12—N1—N2—C14	105.6 (2)
C6—C8—C9—C10	2.3 (3)	C11—N1—N2—C14	−85.1 (2)
O1—C8—C9—C11	3.0 (3)	O6—C21—N3—C19	−1.2 (4)
C6—C8—C9—C11	−176.62 (16)	O6—C21—N3—C20	−178.0 (2)
C8—C9—C10—O2	−2.8 (3)	C9—C10—O2—C5	1.0 (3)
C11—C9—C10—O2	176.18 (16)	C6—C5—O2—C10	1.1 (3)
C10—C9—C11—N1	127.95 (18)	C4—C5—O2—C10	−178.26 (16)
C8—C9—C11—N1	−53.1 (2)	C16—C15—O5—C18	−0.1 (2)
C10—C9—C11—S1	−113.90 (17)	C14—C15—O5—C18	−177.62 (15)
C8—C9—C11—S1	65.1 (2)	C17—C18—O5—C15	0.0 (2)
O3—C12—C13—S1	−171.45 (16)	C12—C13—S1—C11	−13.78 (14)
N1—C12—C13—S1	8.17 (19)	N1—C11—S1—C13	15.17 (13)
O4—C14—C15—C16	−177.9 (2)	C9—C11—S1—C13	−108.82 (14)

### Hydrogen-bond geometry (Å, °)

**Table:**

*D*—H···*A*	*D*—H	H···*A*	*D*···*A*	*D*—H···*A*
N2—H2···O6	0.77 (2)	2.10 (2)	2.856 (2)	169 (2)
C4—H4···O6^i^	0.93	2.50	3.421 (3)	172
C10—H10···O4^ii^	0.93	2.49	3.332 (2)	151
C11—H11···O5^ii^	0.98	2.50	3.267 (2)	135 (1)
C13—H13B···O3^iii^	0.97	2.55	3.441 (2)	153
C16—H16···O6	0.93	2.36	3.193 (3)	150
C18—H18···O3^iv^	0.93	2.47	3.342 (3)	157
C20—H20C···O1^v^	0.96	2.46	3.361 (3)	157

Symmetry codes: (i) −*x*, −*y*+1, −*z*+1; (ii) −*x*, −*y*+2, −*z*+1; (iii) −*x*+1, −*y*+2, −*z*; (iv) −*x*+1, −*y*+2, −*z*+1; (v) −*x*+1, −*y*+1, −*z*.
